# Hand hygiene improvement or antibiotic restriction to control the household transmission of extended-spectrum β-lactamase-producing *Escherichia coli*: a mathematical modelling study

**DOI:** 10.1186/s13756-020-00803-9

**Published:** 2020-08-21

**Authors:** Lidia Kardaś-Słoma, Yazdan Yazdanpanah, Anne Perozziello, Jean-Ralph Zahar, François-Xavier Lescure, Anthony Cousien, Jean-Christophe Lucet

**Affiliations:** 1 INSERM, Infection, Antimicrobials, Modelisation, Evolution (IAME), UMR 1137, F-75018 Paris, France; 2 University of Paris Diderot, IAME, UMR 1137, Sorbonne Paris Cité, F-75018 Paris, France; 3 AP-HP, Bichat-Calude Bernard Hospital, F-75018 Paris, France; 4 AP-HP, Bichat-Claude Bernard Hospital, Infectious and Tropical Diseases Unit, F-75018 Paris, France; 5 AP-HP, Avicenne University Hospital, Infection Control Unit, F-93000 Bobigny, France; 6 AP-HP, Bichat-Claude Bernard Hospital, Infection Control Unit, F-75018 Paris, France

**Keywords:** Extended-spectrum β-lactamase-producing *E. coli*, Household, Control, Mathematical model

## Abstract

**Background:**

The best strategy to control ESBL-producing *Escherichia coli* (ESBL-EC) spread in the community is lacking.

**Methods:**

We developed an individual-based transmission model to evaluate the impact of hand hygiene (HH) improvement and reduction in antibiotic use on the within-household transmission of ESBL-EC**.** We used data from the literature and incorporated key elements of ESBL-EC transmission such as the frequency and nature of contacts among household members, antibiotic use in the community and hand hygiene behaviour. We introduced in a household a single ESBL-EC colonised person and simulated the transmission dynamics of ESBL-EC over a one-year time horizon.

**Results:**

The probability of ESBL-EC transmission depended on the household composition and the profile of the initial carrier. In the two-person household, the probability of ESBL-EC transmission was 5.3% (95% CI 5.0–5.6) or 6.6% (6.3–6.9) when the index person was a woman or a man, respectively. In a four-person household, the probability of transmission varied from 61.4% (60.9–62.0) to 68.8% (68.3–69.3) and was the highest when the index patient was the baby. Improving HH by 50% reduced the probability of transmission by 33–62%. Antibiotic restriction by 50% reduced the transmission by 2–6%.

**Conclusions:**

The transmission of ESBL-EC is frequent in households and especially those with a baby. Antibiotic reduction had little impact on ESBL-EC. Improvement of hygiene in the community could help prevent transmission of ESBL-EC.

## Introduction

Over recent years, the prevalence of extended-spectrum β-lactamase-producing *Enterobacteriaceae* (ESBL-PE) has increased worldwide [1]. Initially, ESBL-PE spread in hospital environments, but currently community-acquired infections caused by ESBL *E. coli* (ESBL-EC) are also on the rise [2].

Community spread of ESBL-EC is an important public health threat for several reasons: gut colonisation amplifies the reservoir of community pathogens and the introduction of resistance into hospitals; infections caused by ESBL-EC lead to inadequate antibiotic therapy; and ESBL-EC infections are associated with consumption of last resort antibiotics, favouring the emergence of carbapenem-resistant *Enterobacteriaceae*.

The prevalence of gut colonisation of ESBL in the community is variable, with carriage rates exceeding 50% in Southeast Asia and the Indian sub-continent, with likely several billion ESBL carriers worldwide. In contrast, in North America and Western Europe, the rate of ESBL carriage is estimated at 5–10% [2].

The sources of ESBL-EC transmission in the community remain controversial. Some studies have shown that after hospital discharge, colonised patients can transmit bacteria to their household [3, 4]. Other studies report frequent acquisition of ESBL-PE during international travel to high-endemic countries [5, 6]. It has also been suggested that acquisition of resistant bacteria in the community may occur from the environment, animals or through the food chain [7, 8].

In a recent study, Mughini-Gras et al. [9] modelled the relative contributions of several sources to community-acquired EBSL-EC carriage in the Netherlands. They found that approximately two-thirds of community-acquired ESBL-EC carriage was attributable to human-to-human transmission, with the non-human sources (food, animal, and environmental) accounting for the other third.

The mechanisms of human-to-human transmission of ESBL-EC in the community are not well understood. However, the household may play an important role in the spread of ESBL-EC due to the proximity of contacts, the sharing of similar exposures and risk factors and multiple opportunities for cross-transmission among household members [4, 10].

Improvement in compliance with hand hygiene (HH) and reduction of antibiotic use are two main control measures for reducing the burden of resistant bacteria in the hospital environment. However, there is little evidence that these measures are effective in preventing the spread of ESBL-EC in the community.

Mathematical models have long been used to study pathogen dissemination in hospitals and to evaluate infection control strategies. Community models of resistance spread and control are scarce, and are often limited to MRSA [11, 12]. Moreover, most of these models were simplified, for example, by ignoring the complex interplay between disease transmission and individual-level risk factors, such as age, patient treatment or the structure of contact networks. Such limitations may be overcome using stochastic agent-based simulations, as illustrated in the study of pandemic influenza or HIV transmission [13, 14].

In this study, we used an agent-based model of pathogen transmission in a hypothetical household to investigate the impact of HH improvement and reduction of antibiotic use on the dynamics of household transmission of ESBL-EC.

## Methods

### Transmission model

We developed an agent-based model of person-to-person transmission in a hypothetical household using *NetLogo* (v 6.0.2) software [15]. We introduced in a family a single ESBL-EC colonised person and simulated the transmission dynamics of ESBL-EC and control interventions over a one-year time horizon. We studied four households: two adults, two adults and a child, two adults and a baby, and two adults and a child and baby (so families of 2–4 persons).

The oral-faecal route was indicated as the most frequent route of human-to-human ESBL-EC transmission [9]. Thus, in the model, we hypothesised that hand contamination with ESBL-EC most likely occurs when: 1) a colonised person is using the toilet or 2) a person is changing the diapers of a colonised baby. Non-human sources may also represent a reservoir of ESBL-EC for humans and contribute to the spread of resistance in the community (e.g. by contaminated meat/vegetables, pets or the environment). By simplification we included in the model a single parameter representing the background acquisition, based on a recent study [3].

Cross-transmission among individuals occurred via contaminated hands. We assumed that feeding a baby or eating with contaminated hands could lead to gut colonisation. If HH was performed after using the toilet/changing diapers and before eating/feeding, it prevented ESBL-EC contamination and colonisation, respectively.

The structure of contact patterns is highly associated with age and gender [16, 17]. In order to infer the contact network in a modelled household and routes of human-to-human transmission, we considered four profiles of household members: adult woman, adult man, child (≥ 3 years old) and baby wearing diapers (< 3 years). For simplification, other household members (relatives, visitors, etc.) were not considered in the model. We modelled each profile explicitly; it had its own contact frequency with household members, level of HH compliance, and exposure to antibiotics.

Each individual could be in one of four infectious states: 1) susceptible (negative for ESBL-EC), 2) contaminated (hands), 3) colonised (in the digestive tract) or 4) colonised and contaminated. The probability of changing the state for each person was updated daily, and depended on the nature and frequency of contacts, ESBL-EC infectious state of household members, HH compliance, and antibiotic exposure.

We hypothesised that exposure to antibiotics may facilitate the transmission of ESBL-EC in two ways: by increasing the probability of colonisation in contaminated persons receiving antibiotics, and by increasing the probability of transmission from a colonised person treated with antibiotics [18].

**Fig. 1:**
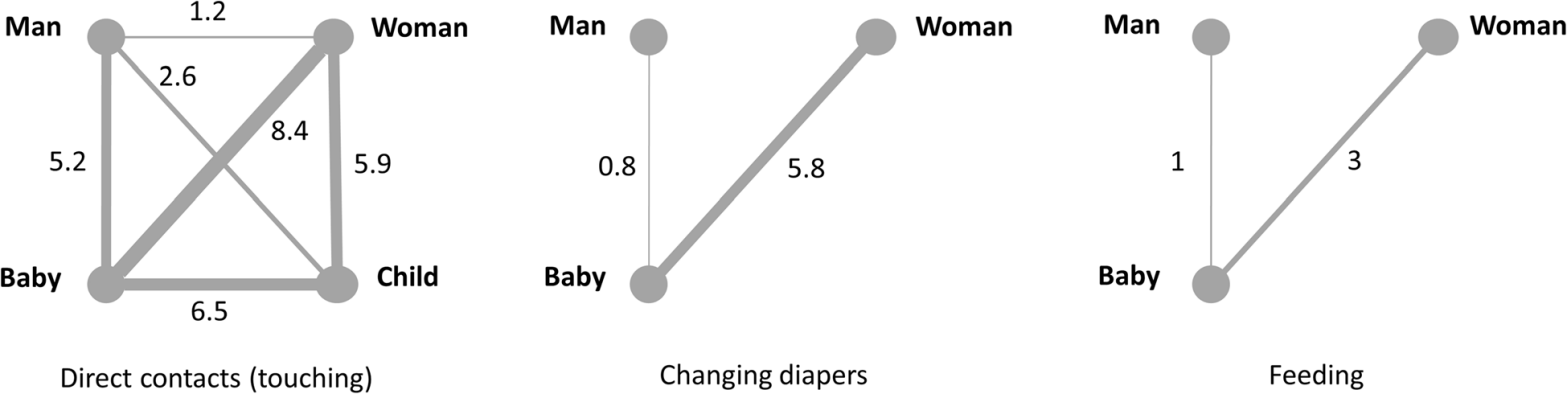
Daily frequency of contacts via touching, changing diapers and feeding within a household. Based on data from **[**17**]**

We derived parameter estimates including daily contacts (Fig. 1), HH practices (Table 1), and other model inputs from the literature (Supplementary Table S1).

**Table 1:** Probability of handwashing with soap in most critical situations for ESBL-EC transmission. Based on data from [17]. ^a^(%) of HH opportunities

	HH after using the toilet (%)^a^	HH before meals (%)^a^	HH after changing diapers (%)^a^	HH before feeding (%)^a^
Woman	40	36	60	0
Man	17	33	50	0
Child	29	50	–	–

Based on French data on antibiotic use in the community, the daily probability of antibiotic prescription was higher for children and babies [19].

There is a lack of data about the probability of hand contamination with ESBL-EC after changing diapers or using the toilet in households. We hypothesised that the probability of hand contamination was higher after changing diapers than after person-to-person contact. For the probability of contamination after using the toilet, we undertook a conservative assumption that it would be the same as for the contact with contaminated hands. In a supplementary analysis, we studied the impact of our assumptions on main results.

An unknown parameter, the daily probability of gut colonisation in a contaminated person (*p*_*col*_), was calibrated in order to reproduce the transmission rate estimated in the study of Arcilla et al. [6].

A detailed description of the model, the main model parameters and details on parameters calibration can be found in the Supplementary Text S1.

### Infection control strategies

In the base case scenario, with no intervention, we considered compliance with HH and antibiotic exposure reported in the literature (Table 1 and Supplementary Table S1). Then, we assessed two scenarios with implementation of control strategies: (1) 50% improvement in compliance with HH for all HH opportunities, (2) antibiotic restriction, with a 50% reduction of patients receiving antibiotics and 25% reduction of treatment duration.

### Model simulations and outcomes

The main outcome of interest was the probability of ESBL-EC transmission to a household member during one year following the introduction of an index case. We also estimated the mean time of persistence of ESBL-EC colonisation, defined as the time it takes to get rid of bacteria from all household members. The outcomes were estimated from 30,000 simulations of the stochastic model for each set of parameter values.

### Univariate uncertainty analysis

The confidence intervals presented in the main analysis reflect the uncertainty due to the stochastic processes of the model and not that associated with uncertainty in parameter estimates. To assess the impact of the latter on our results, we performed several uncertainty analyses. We tested the impact of a lower duration of intestinal colonisation, a lower duration of hand contamination with ESBL-EC, a higher base case level of HH after using the toilet and before eating, a higher probability of hand contamination after using the toilet, and a lower probability of hand contamination after changing diapers (Supplementary Text S2).

We then studied the impact of a higher probability of acquisition from external sources (Supplementary Text S2).

We also investigated the model in which the daily frequency of contacts between man and woman was higher than the reported 1.2 contacts/day to take into account that contacts between man and woman may be less frequent than reported but may last longer (e.g., by sleeping in the same bed).

We tested a less than 50% improvement in HH to take into account the difficulty of improving HH in the community or that the quality of HH needed to eradicate bacteria from hands may be lower than the assumed 100%.

Finally, we investigated the impact of higher, 62% reduction in antibiotic use to reach the lowest European level of antibiotic use in the community, observed in the Netherlands [20].

### Sensitivity analysis

We ran a multivariate sensitivity analysis to quantify the impact of input parameters on the model output. These parameters included epidemiological parameters (probability of contamination after using the toilet/changing diapers, probability of colonisation, duration of colonisation etc.); and transmission control parameters, i.e. compliance with HH and probability of antibiotic prescription. For this analysis, we considered a household composed of two persons or four persons and a woman as an index case. We used Latin Hypercube Sampling (LHS) to generate *N* = 100 parameter sets from our parameter ranges (Supplementary Table S1). For each set of model parameters, we calculated *N* model outputs (over 30,000 simulation replicates). Then, we used the Partial Rank Correlation Coefficient (PRCC) to quantify and rank the impact of input parameters on the probability of ESBL-EC transmission in a household (Supplementary Text S1).

## Results

### Base case scenario

**Fig. 2:**
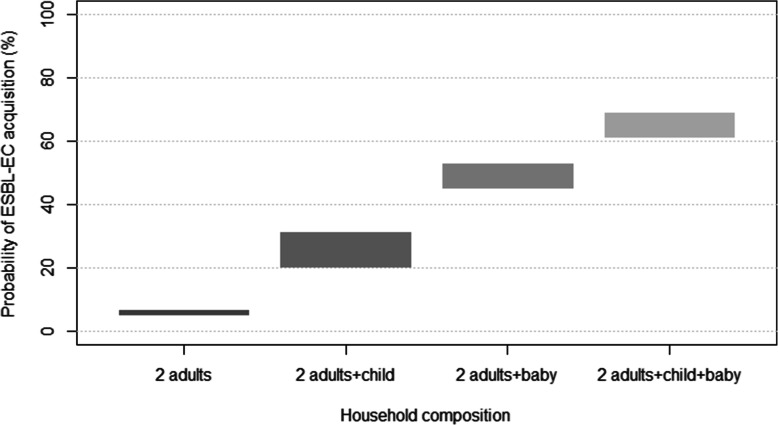
Probability of EBSL-CE transmission over one year in the household according to the family composition and a profile of initial carrier

In the household composed of two adults, over one year, the probability of ESBL-EC transmission was 5.3 and 6.6% when the index person was a woman or a man, respectively (Fig. 2 and Table 2). The probability of transmission depended on the household composition and the profile of the initial carrier. In the household composed of two adults and a child, the probability of ESBL-EC transmission varied from 20.4 to 31.2% and was the highest when the initial carrier was the child. In the household composed of two adults and a baby, the probability of transmission ranged from 45.4 to 52.8% and was the highest when the initial carrier was the baby. Finally, in the household composed of four persons, the probability of ESBL-EC transmission varied from 61.4 to 68.8% and was the highest when the index patient was the baby.

**Table 2:** Probability of ESBL-EC transmission over one year in the household

Control strategy	Household composition^a^	Probability of ESBL-EC transmission according to the profile of initial carrier (%) [95% CI]				Reduction from the base case (%)			
		woman	man	child	baby	woman	man	child	baby
**Base case (A)**	b0c0	5.3 [5.0–5.6]	6.6 [6.3–6.9]	–	–	–	–	–	–
	b0c1	21.4 [21.0–21.9]	20.4 [20.0–20.9]	31.2 [30.6–31.7]	–	–	–	–	–
	b1c0	51.6 [51.1–52.2]	45.4 [44.8–46.0]	–	52.8 [52.2–53.4]	–	–	–	–
	b1c1	65.8 [65.2–66.3]	61.4 [60.9–62]	67.8 [67.36–68.4]	68.8 [68.3–69.3]	–	–	–	–
**HH improvement by 50% (B)**						**(B-A)/A**			
	b0c0	2.8 [2.6–3.0]	4.3 [4.0–4.5]	–	–	−47.2	−34.8	–	–
	b0c1	10.2[9.8–10.5]	12.2 [11.8–12.6]	20.8 [20.3–21.2]	–	−52.3	− 40.2	− 33.3	–
	b1c0	24.5 [24.0–25.0]	26.6 [26.1–27.1]	–	20.1 [19.7–20.6]	− 52.5	− 41.4	–	−61.9
	b1c1	34.5 [34.0–35.1]	38.2 [37.7–38.8]	44.2[43.6–44.8]	28.2 [27.7–28.7]	−47.6	−37.8	−34.8	−59.0
**Reduction of antibiotic use (C)**						**(C-A)/A**			
	b0c0	5.0 [4.7–5.2]	6.3 [6.0–6.5]	–	–	−5.7	−4.5	–	–
	b0c1	20.9 [20.5–21.4]	19.9 [19.4–20.4]	30.2 [29.7–30.7]	–	−2.3	−2.5	−3.2	–
	b1c0	50.2 [49.7–50.8]	43.8 [43.3–44.4]	–	51.3 [50.7–51.8]	−2.7	−3.5	–	− 2.8
	b1c1	63.9 [63.3–64.4]	59.0 [58.4–59.5]	65.9 [65.4–66.4]	67.0 [66.5–67.5]	−2.9	−3.9	−2.8	− 2.6

^a^*b0c0–2 adults without children, b0c1–2 adults + child, b1c0–2 adults + baby, b1c1–2 adults + child + baby*

The mean persistence time of ESBL-EC colonisation was 114.6 days in a household composed of two adults, 127.6 days in a household composed of two adults and a child, 157.1 days in a household composed of two adults and a baby and 188.6 days in a household composed of four persons.

### Implementation of control interventions

Improving HH compliance by 50% was the most effective control intervention to reduce the probability of transmission in a household. The effectiveness depended on the category of initial carrier. The observed reduction from the base case ranged from 47.2 to 52.5% when the initial carrier was a woman, from 34.8 to 41.4% when the initial carrier was a man, from 33.3 to 34.8% when the initial carrier was a child, and from 59 to 61.9% when the initial carrier was a baby (Table 2).

HH improvement by 50% also reduced the mean persistence time of ESBL-EC colonisation in comparison with the base case, with a reduction ranging from 1.5 days in a two-person household to 47.2 days in a four-person household (Table 3).

**Table 3:** Persistence time of ESBL-EC colonisation according to the household composition. Results presented for the household where the initial carrier was the woman

Control strategy	Household composition^a^	Persistence time of ESBL-EC colonisation according to the household composition		
		Mean (days)	SD	Reduction from base case (days)
**Base case**	b0c0	114.6	19.5	–
	b0c1	127.6	40.4	–
	b1c0	157.1	67.3	–
	b1c1	188.6	87.1	–
**HH improvement by 50%**	b0c0	113.1	15.9	−1.5
	b0c1	119.0	28.4	−8.6
	b1c0	129.3	40.6	−27.8
	b1c1	141.4	54.5	−47.2
**Reduction of antibiotic use**	b0c0	114.3	18.7	−0.3
	b0c1	127.1	39.8	−0.5
	b1c0	155.4	65.9	−1.7
	b1c1	184.4	84.8	−4.2

^a^*b0c0–2 adults without children, b0c1–2 adults + child, b1c0–2 adults + baby, b1c1–2 adults + child + baby*

Restricting antibiotic consumption reduced from the base case the probability of transmission in a household from 2.3 to 5.7% when the initial carrier was a woman, from 2.5 to 4.5% when the initial carrier was a man, from 2.8 to 3.2% when the initial carrier was a child, and from 2.6 to 2.8% when the initial carrier was a baby (Table 2). The estimated reduction in persistence time varied from 0.3 to 4.2 days (Table 3).

### Univariate uncertainty analysis

Results of univariate uncertainty analysis for the lower duration of intestinal colonisation, the lower duration of hand contamination with ESBL-EC, the higher base case level of HH, the higher probability of hand contamination after using the toilet, and the lower probability of contamination after changing diapers are presented in Supplementary Text S2.

Higher probability of background acquisition had a little impact on the probability of ESBL-EC transmission to household members (Supplementary Figure S1A). However, with very high values of background acquisition, the transmission originating from household members decreased, the persistence time of ESBL-EC colonisation increased and the impact of HH was limited, indicating that the environment became the most important source of household acquisition (Supplementary Figure S1B).

When the number of daily contacts between man and woman was increased to 5.9 per day (vs. 1.2 in the base case analysis), the probability of ESBL-EC transmission increased for all household compositions, with the highest, more than twice higher, in a household composed of two persons (Supplementary Table S7).

If the impact of HH on the probability of ESBL-EC transmission was lower than in our main analysis (10% vs. 50%), the superiority of the intervention targeting HH compliance over reduction in antibiotic use was confirmed (Supplementary Table 8).

High reduction (by 62%) in antibiotic consumption had a lower impact on the dynamics of ESBL-EC transmission than even a 10% improvement in HH (Supplementary Table S9).

### Sensitivity analysis

We calculated PRCCs between each input parameter and the probability of ESBL-EC transmission, to quantify the importance of inputs for the model output (Table 4). Positive PRCCs indicated that when the value of particular parameter increased, the probability of transmission increased. Negative PRCCs indicated that when the value of particular parameter increased, the probability of transmission in the household decreased. We showed that following parameters were most critical in affecting the output: duration of ESCB-EC colonisation (*d*_*col*_), probability of hand contamination after using the toilet (*p*_*cont,t*_), probability of contact-to-contact transmission (*p*_*tr*_), probability of gut colonisation (*p*_*col*_), HH after using the toilet by the initial carrier (*HH*_*t,w*_).

**Table 4:** Estimates of partial rank correlation coefficients (PRCCs) between the input values of the model and the output of interest, namely the probability of ESBL-EC transmission in the household. ^a^ Not statistically significant

Parameter	Description	PRCC	
		2-person household (index = woman)	4-person household (index = woman)
*d*_*col*_	median duration of colonisation	0.81	0.88
*p*_*cont,t*_	probability of hand contamination after using the toilet	0.81	0.74
*p*_*tr*_	probability of contact-to-contact transmission	0.90	0.76
*p*_*col*_	probability of gut colonisation	0.83	0.61
*rr*_*col*_	relative risk of gut colonisation in a contaminated person receiving antibiotics	0.06^a^	0.50
*p*_*col,feed*_	probability of gut colonisation in a baby	–	0.66
*HH*_*t,w*_	HH after using the toilet, woman	−0.70	−0.58
*HH*_*e,m*_	HH before eating, man	−0.48	− 0.19^a^
*HH*_*f,w*_	HH before feeding, woman	–	−0.44
*HH*_*e,ch*_	HH before eating, children	–	−0.30

## Discussion

This study is the first to investigate the impact of HH improvement and reduction of antibiotic use on the dynamics of within-household transmission of ESBL-EC. Our results underline the importance of HH and the little impact that antibiotic reduction has on the dynamics of ESBL-EC transmission. Improving HH compliance by 50% reduced the probability of ESBL-EC transmission by 30 to 60% according to the household composition and the category of index carrier. One reason that HH was the most effective control measure was that it acts in three ways, i.e. preventing contamination after contact with potentially ESBL-positive faecal matter; accelerating the spontaneous decontamination of hands and preventing cross-transmission of contamination; and preventing gut colonisation from contaminated hands.

HH with soap is a highly effective means of reducing infectious disease transmission; a systematic review showed that HH reduces the risk of diarrhoea episodes by 42–47% [21] and reduces the rate of respiratory infections by 5–34% [22]. Although the importance of HH in preventing infections is obvious, compliance in the community remains low. A systematic review showed that approximately 19% of the world population washes their hands with soap after contact with faeces (13–17% in low- and middle-income regions, and 46–49% in high-income regions) [23].

We searched PubMed to review studies focusing on the household prevalence of HH with soap in the key risk moments for infection transmission. Most publications concerned developing countries and studies from developed countries mainly reported HH frequency after contact with excreta [23]. In our model, we used data on HH based on the study of Miura et al. [17]. To our knowledge, it was the only report describing in details HH behaviour in households for different categories of household members. Given the high estimated impact of HH on ESBL-EC transmission into the household, further research is urgently needed to develop methods of HH measurement in the community, to assess current household levels of HH and to determine the opportunities for efficient hand hygiene.

Our modelling study showed that the probability of ESBL-EC transmission was higher in households with children and especially those with a baby. Other studies also indicated a unique place of children in the transmission dynamics of ESBL-PE [24]*.* The higher probability of acquisition and transmission could be explained by the intensity of contacts between children and other household members, frequent contacts with contaminated environment and limited HH. Islam et al. found that the prevalence of intestinal carriage of ESBL-PE in U.S. children was the highest in 1 to < 2-year-olds and < 5-year-olds (6.5 and 5.2% vs.1.7% in children over 5 years old). Another study reported the transmission between a child carrying ESBL-EC and their family members in 23% of cases [25]. In our study, the probability of transmission from a child or a baby was estimated at 31.2 or 52.8%, respectively. This could be explained by the long duration of colonisation considered in our model (111 days vs. 36 days (4–60), observed in the cited study). In an additional analysis, we fixed that the duration of gut colonisation at 36 days; this reduced the probability of transmission in a family composed of three persons and was 11.4 or 22.1%, when an index carrier was a child or a baby (Supplementary Text S2).

Human exposure to ESBL-EC may occur via raw meat, vegetables, animals, the environment, and human-to-human transmission. In particular, a high prevalence of ESBL-EC has been reported in retail chicken meat; however their role as a main cause of human EC infections remains controversial [8]. In a recent study, Mughini-Gras et al. quantified the significance of different sources of community-acquired ESCB-EC colonisation [9]. They indicated humans as the most important cause; however, the other sources also represented a large reservoir of ESBLs. In our model, we included the daily probability of ESBL-EC background acquisition from a recent study and based on a specific epidemiological situation in Dutch population [3]. This value was much lower that the probability of colonisation due to cross-transmission considered here. Thus, in an additional analysis, we investigated the impact of an increased probability of background acquisition on model predictions. We found that if the probability of background acquisition increased, it had a major impact on the persistence of colonisation in households, limited the impact of HH, and thus may subsequently contribute to community transmission.

Antibiotic use and misuse are the major forces associated with selection of resistant bacteria. However, reducing antibiotic use in the community gave divergent results on the reversion of antibiotic resistance. Indeed, studies examining the impact of antibiotic restriction on resistance were mostly performed in hospital settings and extrapolation from the hospital to the community is not straightforward [26]. Few studies have investigated the impact of antibiotic reduction on the resistance of *E. coli* in the community. One showed that a 28% reduction in the overall use of quinolones resulted in a significant increase in the susceptibility of *E. coli* to quinolones [26]. Another showed that antibiotic stewardship led to reduction of ciprofloxacin and cephalosporins and decreased the incidence of infections caused by ESBL-EC in the community [27]. One other study investigated the impact of restriction of sulphonamide prescription in the UK on the prevalence of resistance in *E. coli* [28]. Although the number of prescriptions decreased by 98% from 1991 to 1999, the frequency of *E. coli* resistance to sulphamethoxazole increased from 39.7 and 46%.

In our study, we compared the effectiveness of antibiotic reduction with improvement of HH in the community. Our results showed that an optimistic scenario with 50% restriction in antibiotic use reduced the probability of transmission modestly, by 2–6%, and that even a 10% improvement of HH compliance was more effective than a 62% reduction in antibiotic use in the community. Although antibiotic stewardship programmes may be important [27], our results show that improvement of HH was more effective in controlling the transmission of ESBL-EC in the community.

Our study has several limitations. Firstly, there are several uncertainties surrounding input parameters. In particular the probability of hand contamination with ESBL-EC after changing diapers or the probability of cross-transmission were inferred from a hospital study and an in-vitro study [29, 30] that are not representative of real-life community settings. Moreover, non-human sources of ESBL-EC acquisition were represented here by a single parameter and originally calculated for a low-prevalence country [3]. Further research is needed to provide better estimates of these important inputs of the model. Secondly, the impact of indirect contact routes (e.g. cleaning cloths and hand contact surfaces such door handles, tap handles, etc.) in the ESBL-EC transmission in households is not well studied and thus not considered here. Thirdly, by simplification, we considered HH opportunities after using the toilet/changing diapers and before eating/feeding. Adding more opportunities (e.g. before cooking, after handling raw food) might increase the rate of contamination and reduce the benefits observed from potential public health intervention. Moreover, we did not model individual comorbidities, old age, previous hospital stays or travelling to endemic countries that may influence the transmission dynamics of ESBL-PE in the community. Finally, we based our predictions on data from developed countries. Further research is needed to study the impact of HH on the transmission of ESBL-EC in developing countries where access to sanitation is limited and the probability of direct contamination from the environment could be very high.

The major strength of our study is the use of an individual-based model that incorporates key but still rare elements of the transmission dynamics of ESBL-EC, such as the frequency and nature of contacts among household members, impact of antibiotic treatment and HH. Furthermore, it was calibrated on actual data. Secondly, we quantified the effectiveness of antibiotic reduction and the improvement of HH in the community which would be very difficult to implement and compare in an interventional study. Finally, we used sensitivity analyses to assess the impact of uncertain input parameters on the outcomes of interest and to identify parameters to prioritise in future research. These parameters should be carefully documented if modelling studies are to guide policies regarding infection control measures.

In conclusion, our model findings suggest that the probability of ESBL-EC transmission is high in households and especially those with a baby. Improving HH was the most effective intervention to reduce the spread of ESBL-EC in the community, as compared to antibiotic reduction. Major efforts should be directed towards improving hygiene in the community in order to limit the spread of ESBL-EC.

## Supplementary information

**Additional file 1.**

## Data Availability

All data generated or analysed during this study are included in this article.

## Abbreviations

ESBL-PE: extended-spectrum β-lactamase-producing *Enterobacteriaceae*
ESBL-EC: ESBL-producing *Escherichia coli*
HH: hand hygiene
