# Supplementary material for: Hand hygiene improvement or antibiotic restriction to control the household transmission of extended-spectrum β-lactamase-producing Escherichia coli: a mathematical modelling study
Source: Antimicrob Resist Infect Control. 2020 Aug 21;9:139. doi: 10.1186/s13756-020-00803-9 (PMC7441222; doi:10.1186/s13756-020-00803-9)
Supplement: Supplementary file 1 — Additional file 1. [file 13756_2020_803_MOESM1_ESM.docx]

**Supplementary Text S1**

**Transmission model**

We developed a stochastic agent-based, discrete-time model, applied to simulate the transmission of ESBL-EC among household members. Individuals are characterized by several variables; e.g., class age (adults, children≥ 3 years old, babies < 3 years ), exposition to antibiotics, number of contacts with family members and hand hygiene behavior. Each individual may be in one of four infectious states: 1) susceptible (negative for ESBL-EC), 2) contaminated (hands), 3) colonised (in the digestive tract) or 4) colonised and contaminated.

The model uses a time step of one day. After the introduction of a colonized person, and then, for each colonized, there is a daily probability of hands contamination by using the toilet (or if an index person is a baby, by changing diapers). This probability *P_cont_* depends on the daily frequency of using the toilet/changing diapers, *c_cont_*, probability of hand contamination after contact with the excrement (*p_cont_*), and compliance with hand hygiene after contact with the excrement (*hh_cont_*).

*P_cont_*= [1 – (1- *p_cont_*) *^ccont^* ]* (1-*hh_cont_*), where the term 1 – (1- *p_cont_*) *^ccont^* describes the probability of becoming contaminated in *c_cont_* trials and represents a cdf of the geometric distribution.

On each day of the simulation, contaminated individuals come into contact with other family members, and then the cross-transmission of the pathogen could occur.

The probability of cross-transmission depends on the number of daily contacts with a family members (*c*) and the probability of pathogen transmission during contact (*p_tr_*).

*P_tr_=* [1 – (1- *p_tr_*) *^c^*].

Once a person is contaminated, he may become colonized (or colonize a baby by feeding) with a probability that depends on the daily frequency of feeding a baby/eating (c*_col_*), probability of hand hygiene before feeding/eating (hh*_col_*) and the probability of gut colonization (p*_col_*).

*P_col_=*[1 – (1- *p_col_*) *^ccol^* ]* (1-*hh_col_*)

Exposure to antibiotics may facilitate the transmission of ESBL-EC in two ways: 1) increase the probability of transmission from colonized person treated with antibiotics (by the relative risk of contamination *rr_cont_*) and 2) increase the probability of colonisation in contaminated persons receiving antibiotic (by the relative risk of colonization *rr_col_*).

During the simulation, when an individual enters a new infectious state, the duration for which he will remain in this state is drawn from a Gamma distribution, with a mean value set by the user.

**Model calibration**

For the unknown parameter, the daily probability of gut colonization in a contaminated person (*p_col_)*, we tested a range of values from 0 to 1. For each combination of model parameters with *p_col_*, we ran 30 000 Monte Carlo simulations. Then, using a least square criterion, we calibrated the *p_col_* in order to best reproduce observed person-to-person transmission rate from the study of Arcilla et al. [1].

**Main model parameters**

**Supplementary Table S1.** Base case values of input parameters used in the model of ESBL-EC transmission in a household and ranges for the sensitivity analysis.

| **Parameters** | **Description** | **Base case values** | **Sources** | **Range tested for the sensitivity analysis** |
| --- | --- | --- | --- | --- |
|  | **Population** |  |  |  |
| *N_h_* | Household size | 2-4 | assumed |  |
|  | **Antibiotic treatment** |  |  |  |
|  | Daily probability of antibiotic prescription : |  |  |  |
| *p_atb,m_*  *p_atb,w_*  *p_atb,ch_*  *p_atb,bb_* | Men  Women  Children  Babies | 2.86 * 10^-3^  2.86 * 10^-3^  5.30 * 10^-3^  8.52 * 10^-3^ | [2] | 1.50* 10^-3^ – 4.5* 10^-3^  1.50* 10^-3^ – 4.5* 10^-3^  2.65* 10^-3^ –7.95* 10^-3^  4.26* 10^-3^ – 1.28* 10^-2^ |
| *d_atb_* | Duration of treatment (days) | 6 | [3] | 5-8 [3] |
|  | **Contamination during person-to-person contact** |  |  |  |
| *p_tr_* | Probability of contamination (per contact) | 0.1 | [4,5] | 0.05- 0.35[4,5] |
| *d_cont_* | Duration of contamination (independent of HH)(hours) | 24 | [6] | 1-24[6] |
|  | **Contamination by changing diapers** |  |  |  |
| *p_cont,d_* | Probability of hand contamination during changing diapers of a colonized baby not receiving antibiotics (per change) | 0.3 | Assumed 3 times higher than after contact with a contaminated person | 0.1-1 [7] |
| *rr_cont_* | Relative risk of hand contamination during changing diapers from a colonized baby receiving antibiotics | 3 | [8,9] | 2-9[8–10] |
|  | **Contamination by using the toilet** |  |  |  |
| *p_cont,t_* | Probability of hand contamination after using the toilet by a colonized person not receiving antibiotics (per toilet use) | 0.1 | Assumed the same as after contact with a contaminated person | 0.05- 0.35[4,5] |
| *rr_cont_* | Relative risk of hand contamination after using the toilet by a colonized person receiving antibiotics | 3 | [8,9] | 2-9[8–10] |
|  | **Colonisation** |  |  |  |
| *p_col_* | Daily probability of gut colonisation in a contaminated person | 0.012 | Calibrated to reproduce the transmission rate from [1] | 0.006-0.018 |
| *p_col,feed_* | Daily probability of gut colonization in a baby feeded by a contaminated person | 0.012 | Assumed the same as  *p_col_* |  |
| *rr_col_* | Relative risk of gut colonisation in a contaminated person receiving antibiotics | 3 | [11–16] | 1.2-15 [11–16] |
| *d_col_* | Duration of colonisation (days) | 111 | [17] | 55-217 ^14^ |
|  | **Background acquisition** |  |  |  |
| *p_col,env_* | Daily probability of background acquisition | 1.5*10^-4^ | ^14^ | 2*10^-5^ – 9*10^-4^ [1,17] |
|  | **Hand hygiene after changing diapers** |  |  |  |
| *HH_d,w_* | HH among women | 0.6 | [18] | 0.3-0.9 |
| *HH_d,m_* | HH among men | 0.5 | [18] | 0.25-0.75 |
|  | **Hand hygiene after using the toilet** |  |  |  |
| *HH_t,w_* | HH among women | 0.4 | [18] | 0.2-0.6 |
| *HH_t,m_* | HH among men | 0.17 | [18] | 0.085-0.255 |
| *HH_t,ch_* | HH among children | 0.29 | [18] | 0.145-0.435 |
|  | **Hand hygiene before eating** |  |  |  |
| *HH_e,w_* | HH among women | 0.36 | [18] | 0.18-0.54 |
| *HH_e,m_* | HH among men | 0.33 | [18] | 0.165-0.495 |
| *HH_e,ch_* | HH among children | 0.5 | [18] | 0.25-0.75 |
|  | **Hand hygiene before feeding a baby** |  |  |  |
| *HH_f,w_* | HH among women | 0 |  | 0-0.5 |
| *HH_f,m_* | HH among men | 0 |  | 0-0.5 |

**Sensitivity analysis**

We considered *k* model parameters in the sensitivity analysis (*k*=24). Because there was no *a priori* information on parameter distributions, we chose the uniform distribution with minimum and maximum values from given parameter ranges (Supplementary Table S1).

The range of each parameter value was divided into *N* equal probability intervals, which are then sampled (*N*=100 and represents the sample size). The sampling is done by randomly selecting values from each pdf. Each interval for each parameter is sampled exactly once (without replacement), so that the entire range for each parameter is explored. For more details see Marino et al.[19]. We generated a LHS matrix that consists of *N* rows (sample size) and *k* columns corresponding to the number of parameters. Then, for each combination of model parameters, we calculated *N* model outputs (over 30 000 simulation replicates).

We used Partial Rank Correlation Coefficient (PRCC) to quantify and rank the impact of input parameters on the probability of ESBL-EC transmission in a household. For more details see Blower et al.[20].

**References**

1. Arcilla MS, Hattem JM van, Haverkate MR, et al. Import and spread of extended-spectrum β-lactamase-producing Enterobacteriaceae by international travellers (COMBAT study): a prospective, multicentre cohort study. Lancet Infect Dis. **2017**; 17(1):78–85.

2. Sabuncu E, David J, Bernède-Bauduin C, et al. Significant reduction of antibiotic use in the community after a nationwide campaign in France, 2002-2007. PLoS Med. **2009**; 6(6):e1000084.

3. ANSM. L’évolution des consommations d’antibiotiques en France entre 2000 et 2015 [Internet]. 2017 [cited 2019 Jan 15]. Available from: https://ansm.sante.fr/var/ansm_site/storage/original/application/188a6b5cf9cde90848ae9e3419bc3d3f.pdf

4. Pessoa-Silva CL, Dharan S, Hugonnet S, et al. Dynamics of bacterial hand contamination during routine neonatal care. Infect Control Hosp Epidemiol. **2004**; 25(3):192–197.

5. Bellissimo-Rodrigues F, Pires D, Soule H, Gayet-Ageron A, Pittet D. Assessing the Likelihood of Hand-to-Hand Cross-Transmission of Bacteria: An Experimental Study. Infect Control Hosp Epidemiol. **2017**; 38(5):553–558.

6. Scott E, Bloomfield SF. The survival and transfer of microbial contamination via cloths, hands and utensils. J Appl Bacteriol. **1990**; 68(3):271–278.

7. Gibson LL, Rose JB, Haas CN, Gerba CP, Rusin PA. Quantitative assessment of risk reduction from hand washing with antibacterial soaps. J Appl Microbiol. **2002**; 92 Suppl:136S–43S.

8. Cheng VCC, Li IWS, Wu AKL, et al. Effect of antibiotics on the bacterial load of meticillin-resistant Staphylococcus aureus colonisation in anterior nares. J Hosp Infect. **2008**; 70(1):27–34.

9. Wang J, Wang M, Huang Y, et al. Colonization pressure adjusted by degree of environmental contamination: A better indicator for predicting methicillin-resistant Staphylococcus aureus acquisition. Am J Infect Control. **2011**; 39(9):763–769.

10. Lastours V de, Chopin D, Jacquier H, et al. Prospective Cohort Study of the Relative Abundance of Extended-Spectrum-Beta-Lactamase-Producing Escherichia coli in the Gut of Patients Admitted to Hospitals. Antimicrob Agents Chemother. **2016**; 60(11):6941–6944.

11. Colodner R, Rock W, Chazan B, et al. Risk factors for the development of extended-spectrum beta-lactamase-producing bacteria in nonhospitalized patients. Eur J Clin Microbiol Infect Dis Off Publ Eur Soc Clin Microbiol. **2004**; 23(3):163–167.

12. Nicolas-Chanoine M-H, Jarlier V, Robert J, et al. Patient’s origin and lifestyle associated with CTX-M-producing Escherichia coli: a case-control-control study. PloS One. **2012**; 7(1):e30498.

13. Stewardson AJ, Vervoort J, Adriaenssens N, et al. Effect of outpatient antibiotics for urinary tract infections on antimicrobial resistance among commensal Enterobacteriaceae: a multinational prospective cohort study. Clin Microbiol Infect. **2018**; 24(9):972–979.

14. Hilliquin D, Le Guern R, Thepot Seegers V, et al. Risk factors for acquisition of OXA-48-producing Klebsiella pneumonia among contact patients: a multicentre study. J Hosp Infect. **2018**; 98(3):253–259.

15. Carmeli Y, Eliopoulos GM, Samore MH. Antecedent Treatment with Different Antibiotic Agents as a Risk Factor for Vancomycin-Resistant Enterococcus. Emerg Infect Dis. **2002**; 8(8):802–807.

16. Donskey CJ, Chowdhry TK, Hecker MT, et al. Effect of Antibiotic Therapy on the Density of Vancomycin-Resistant Enterococci in the Stool of Colonized Patients. N Engl J Med. **2000**; 343(26):1925–1932.

17. Haverkate MR, Platteel TN, Fluit AC, et al. Quantifying within-household transmission of extended-spectrum β-lactamase-producing bacteria. Clin Microbiol Infect Off Publ Eur Soc Clin Microbiol Infect Dis. **2017**; 23(1):46.e1-46.e7.

18. Miura F, Watanabe T, Watanabe K, Takemoto K, Fukushi K. Comparative assessment of primary and secondary infection risks in a norovirus outbreak using a household model simulation. J Environ Sci China. **2016**; 50:13–20.

19. Marino S, Hogue IB, Ray CJ, Kirschner DE. A methodology for performing global uncertainty and sensitivity analysis in systems biology. J Theor Biol. **2008**; 254(1):178–196.

20. Blower SM, Dowlatabadi H. Sensitivity and Uncertainty Analysis of Complex Models of Disease Transmission: An HIV Model, as an Example. Int Stat Rev Rev Int Stat. **1994**; 62(2):229–243.

**Supplementary Text S2**

**Uncertainty analysis**

1. ***Impact of the lower duration of intestinal colonisation.***

We run an uncertainty analysis with the duration of intestinal colonisation lower than in our main analysis; 36 days (vs 111 at baseline).

***Results***

The estimated probability of ESBL-EC transmission in a 2-persons household was 1.8% and 2.2% when the index person was a woman or a man, respectively (Supplementary Table S1). In the household composed of 2 adults and a child the probability of ESBL-EC transmission varied from 7.2 11.4%. In the household composed of two adults and a baby, the probability of transmission ranged from 18.1 to 22.1%. In the household composed of four persons, the probability of ESBL-EC transmission varied from 26.1 to 31.8%.

| **Household composition*** | **Probability of ESBL-EC transmission according to the profile of the initial carrier (%) [95% CI]** | | | |
| --- | --- | --- | --- | --- |
|  | **woman** | **man** | **child** | **baby** |
| **b0c0** | 1.8 [1.6-1.9] | 2.2 [2.1-2.4] | - | - |
| **b0c1** | 7.6 [7.3-7.9] | 7.2 [6.9-7.5] | 11.4 [11.0-11.8] | - |
| **b1c0** | 21.5 [21.0-21.9] | 18.1 [17.6-18.5] | - | 22.1[21.6-22.5] |
| **b1c1** | 29.6 [29.1-30.2] | 26.1 [25.6-26.6] | 31.0 [30.5-31.5] | 31.8 [31.2-32.3] |

**Supplementary Table S1**. Results of univariate uncertainty analysis where the duration of intestinal colonisation was 36 days (vs 111 days).

*b0c0- 2 adults without children, b0c1- 2 adults+ child, b1c0- 2 adults + baby, b1c1- 2 adults+ child+ baby.

1. ***Impact of the lower duration of hand contamination with ESBL-EC.***

We investigated a modified model, in which the duration of hand contamination with ESBL-EC less than one hour vs less than one day in the central analysis.

***Results***

Lower duration of hand contamination reduced the overall probability of ESBL-EC transmission in a household. In a 2-persons household, it was 2.8% and 3.5% when the index person was a woman or a man, respectively (Supplementary Table S2). In the household composed of 2 adults and a child the probability of ESBL-EC transmission varied from 10.2 to 18.0% and was the highest when the initial carrier was the child. In the household composed of two adults and a baby, the probability of transmission ranged from 25.2 to 40.7% and was the highest when the initial carrier was the baby. In the household composed of four persons, the probability of ESBL-EC transmission varied from 30.0 to 48.2% and was the highest when the index patient was the baby.

**Supplementary Table S2.** Results of univariate uncertainty analysis where the duration of hand contamination with ESBL-EC was less than one hour (vs less than 1 day in the base case analysis).

| **Household composition*** | **Probability of ESBL-EC transmission according to the profile of the initial carrier (%) [95% CI]** | | | |
| --- | --- | --- | --- | --- |
|  | **woman** | **man** | **child** | **baby** |
| **b0c0** | 2.8 [2.6-3.0] | 3.5 [3.3-3.7] | - | - |
| **b0c1** | 10.8 [10.4-11.1] | 10.2 [9.8-10.5] | 18.0 [17.5-18.4] | - |
| **b1c0** | 34.5 [33.9-35.0] | 25.2 [24.7-25.7] | - | 40.7 [40.1-41.2] |
| **b1c1** | 40.4 [39.8-40.9] | 30.0 [29.5-30.5] | 37.0 [36.4-37.5] | 48.2 [47.6-48.8] |

*b0c0- 2 adults without children, b0c1- 2 adults+ child, b1c0- 2 adults + baby, b1c1- 2 adults+ child+ baby.

1. ***Impact of the higher base case level of HH after using the toilet and before eating.***

We studied the impact of the higher base case level of HH:1) after using the toilet at 72%, and 2) before eating at 74% reported recently in the Netherlands’ general population (van den Bunt, 2019).

***Results***

With the higher base case level of HH after using the toilet and before eating, the probability of ESBL-EC transmission was lower (e.g. 1.1% in the two-person household) but still the highest in the households with a baby (19%- 39.1%).

**Supplementary Table S3.** Results of univariate uncertainty analysis where the base case level of HH after using the toilet and before eating was at.72%, and 74% respectively.

| **Household composition*** | **Probability of ESBL-EC transmission according to the profile of the initial carrier (%)**  **[95% CI]** | | | |
| --- | --- | --- | --- | --- |
|  | **woman** | **man** | **child** | **baby** |
| **b0c0** | 1.1 [1.0-1.3] | 1.1 [1.0-1.2] | - | - |
| **b0c1** | 5.7 [5.5-6.0] | 4.1 [3.9-4.3] | 6.3 [6.0-6.6] | - |
| **b1c0** | 29.2 [28.6-29.7] | 19.0 [18.6-19.5] | - | 26.2 [25.7-26.7] |
| **b1c1** | 37.7 [37.2-38.3] | 26.8 [26.3-27.3] | 32.9 [32.3-33.4] | 39.1 [38.6-39.7] |

*b0c0- 2 adults without children, b0c1- 2 adults+ child, b1c0- 2 adults + baby, b1c1- 2 adults+ child+ baby.

1. ***Impact of the higher probability of hand contamination after using the toilet.***

We investigated a modified model, with the higher probability of hand contamination after using the toilet (the same as the probability of hand contamination after changing diapers).

***Results***

Higher probability of hand contamination after using the toilet increased the probability of ESBL-EC transmission in all household, except those where the initial carrier was the baby. The highest probabilities of transmission were observed in households with a baby (b1c0 and b1c1), and ranged from 53 to 84%.

**Supplementary Table S4.** Results of univariate uncertainty analysis where the probability of hand contamination after using the toilet was 0.3 (vs 0.1 in the base case analysis).

| **Household composition*** | **Probability of ESBL-EC transmission according to the profile of the initial carrier (%)**  **[95% CI]** | | | |
| --- | --- | --- | --- | --- |
|  | **woman** | **man** | **child** | **baby** |
| **b0c0** | 9.3 [9.0-9.6] | 10.9 [10.6-11.3] | - | - |
| **b0c1** | 34.1 [33.6-34.7] | 32.0 [31.5-32.5] | 46.5 [46.0-47.1] | - |
| **b1c0** | 70.5 [70.0-71.0] | 62.9 [62.4-63.5] | - | 53.5 [52.9-54.0] |
| **b1c1** | 82.7 [82.2-83.1] | 77.7 [77.2-78.2] | 84 [83.2-84.0] | 68.1 [67.6-68.6] |

*b0c0- 2 adults without children, b0c1- 2 adults+ child, b1c0- 2 adults + baby, b1c1- 2 adults+ child+ baby.

1. ***Impact of the lower probability of hand contamination after changing diapers.***

Lower probability of hand contamination after changing diapers (0.1 vs 0.3) had the impact on the probability of ESBL-EC transmission but only in households where the initial carrier was the baby.

**Supplementary Table S5** Results of univariate uncertainty analysis where the probability of hand contamination after changing diapers was 0.1 (vs 0.3 in the base case analysis).

| **Household composition*** | **Probability of ESBL-EC transmission according to the profile of the initial carrier (%) [95% CI]** | | | |
| --- | --- | --- | --- | --- |
|  | **woman** | **man** | **child** | **baby** |
| **b0c0** | 5.3 [5.0-5.5] | 6.6 [6.3-6.9] | - | - |
| **b0c1** | 21.4 [20.9-21.9] | 20.4 [19.9-20.8] | 31.1 [30.5-31.6] | - |
| **b1c0** | 51.4 [50.8-52.0] | 45.3 [44.7-45.9] | - | 34.9 [34.4-35.4] |
| **b1c1** | 65.2 [64.7-65.7] | 61.2 [60.7-61.8] | 67.1 [66.6-67.6] | 50.2 [49.6-50.7] |

*b0c0- 2 adults without children, b0c1- 2 adults+ child, b1c0- 2 adults + baby, b1c1- 2 adults+ child+ baby.

1. ***Impact of the higher probability of background acquisition and impact of improvement in HH.***

We also investigated the model in which the daily probability of background acquisition (*p_col,env_*) was higher than in our main analysis.

***Results***

Higher probability of background acquisition had a little impact on the probability of ESBL-EC transmission (Supplementary Figure S1A). Moreover, with very high values of background acquisition, the transmission originated from household members decreased, the persistence time of ESBL-EC colonisation increased, and the impact of improved HH by 50% was limited. These results indicated that the environment became the most important source of household acquisition and HH improvement will not be sufficient to limit the spread of ESBL-EC (Supplementary Figure S1B).

**Supplementary Figure S1A**. Probability of ESBL-EC transmission in the model where the daily probability of background acquisition was higher than 0.00015 (base case on red) and the impact of improving HH by 50%. Results presented for the 4-persons household, where the initial carrier was the woman.

**Supplementary Figure S1B**. Persistence time of ESBL-EC transmission in the model where the daily probability of background acquisition was higher than 0.00015 (base case on red) and the impact of improving HH by 50%. Results presented for the 4-persons household, where the initial carrier was the woman.

1. ***Impact of the increased number of daily contacts between man and woman in a household.***

We investigated the model with the daily frequency of contacts between man and woman higher than the reported 1.2 contacts/day to take into account that contacts between man and woman may be less frequent than reported but may last longer (e.g., by sleeping in the same bed).

***Results***

When the number of daily contacts between man and woman was increased to 5.9 per day (vs. 1.2 in the base case analysis), the probability of ESBL-EC transmission increased for all household compositions, with the highest, more than twice higher, in a household composed of 2 persons (Supplementary Table S4).

**Supplementary Table S7.** Results of univariate uncertainty analysis where the daily frequency of contacts between man and woman was 5.9 (vs 1.2 in the base case analysis).

| **Household composition*** | **Probability of ESBL-EC transmission according to the profile of the initial carrier (%) [95% CI]** | | | |
| --- | --- | --- | --- | --- |
|  | **woman** | **man** | **child** | **baby** |
| **b0c0** | 18.6 [18.2-19.0] | 22.0 [21.5-22.4] | - | - |
| **b0c1** | 33.6 [33.1-34.1] | 35.4 [34.9-36.0] | 35.3[34.8-35.9] | - |
| **b1c0** | 62.6 [62.0-63.1] | 66.1 [65.6-66.6] | - | 61.2 [60.6-61.8] |
| **b1c1** | 74.1[73.6-74.6] | 76.8[76.3-77.3] | 73.1 [72.6-73.6] | 74.1 [73.6-74.6] |

*b0c0- 2 adults without children, b0c1- 2 adults+ child, b1c0- 2 adults + baby, b1c1- 2 adults+ child+ baby.

1. ***Impact of the 10% improvement in hand hygiene compliance.***

When the impact of HH on the probability of ESBL-EC transmission was lower than in our main analysis (10% vs. 50%), the superiority of the intervention targeting hand hygiene compliance over reduction in antibiotic use was conﬁrmed (Supplementary Table S5).

**Supplementary Table S8.** Results of univariate uncertainty analysis where the HH improved by 10% (vs 50%).

| **Household composition*** | **Probability of ESBL-EC transmission according to the profile of the initial carrier (%)[95% CI]** | | | | **Reduction from the base case**  **(%)** | | | |
| --- | --- | --- | --- | --- | --- | --- | --- | --- |
|  | **woman** | **man** | **child** | **baby** | **woman** | **man** | **child** | **baby** |
| **b0c0** | 5.0 [4.7-5.2] | 6.1 [5.8-6.3] | - | - | -5.7 | -7.6 | - | - |
| **b0c1** | 19.2 [18.8-19.7] | 19.2 [18.8-19.6] | 28.7 [28.2-29.2] | - | -10.3 | -5.9 | -8.0 | - |
| **b1c0** | 46.7 [46.1-47.2] | 42.4 [41.8-42.9] | - | 47.6 [47.0-48.1] | -9.5 | -6.6 | - | -9.8 |
| **b1c1** | 60.2 [59.6-60.7] | 57.4 [56.8-58.0] | 64.7 [64.1-65.2] | 63.3 [62.7-63.8] | -8.5 | -6.5 | -4.6 | -8.0 |

*b0c0- 2 adults without children, b0c1- 2 adults+ child, b1c0- 2 adults + baby, b1c1- 2 adults+ child+ baby.

1. ***Impact of the 62% reduction in antibiotic use.***

When the antibiotic exposure decreased by 62%, the observed reduction in the probability of ESBL-EC transmission was still lower than even a 10% improvement in HH compliance.

**Supplementary Table S9.** Results of univariate uncertainty analysis where the antibiotic use was reduced by 62%.

| **Household composition*** | **Probability of ESBL-EC transmission according to the profile of the initial carrier (%)[95% CI]** | | | | **Reduction from the base case**  **(%)** | | | |
| --- | --- | --- | --- | --- | --- | --- | --- | --- |
|  | **woman** | **man** | **child** | **baby** | **woman** | **man** | **child** | **baby** |
| **b0c0** | 5.2 [5.1-5.6] | 6.4 [6.2-6.7] | - | - | -1.9 | -3.0 | - | - |
| **b0c1** | 20.9 [20.1-21.0] | 19.7 [19.2-20.1] | 29.6 [29.1-30.1] | - | -2.3 | -3.4 | -5.1 | - |
| **b1c0** | 49.2 [48.7-49.9] | 43.5 [42.9-44.1] | - | 51 [50.4-51.5] | -4.7 | -4.2 | - | -3.4 |
| **b1c1** | 63.1 [62.8-63.9] | 58.6 [58.1-59.2] | 65.7 [65.2-66.3] | 66.2 [65.6-66.7] | -4.1 | -4.6 | -3.1 | -3.8 |

*b0c0- 2 adults without children, b0c1- 2 adults+ child, b1c0- 2 adults + baby, b1c1- 2 adults+ child+ baby.
